# Cancer-mesothelial and cancer-macrophage interactions in the ovarian cancer microenvironment

**DOI:** 10.1152/ajpcell.00461.2022

**Published:** 2023-08-07

**Authors:** Dorota E. Jazwinska, Diana G. Kulawiec, Ioannis K. Zervantonakis

**Affiliations:** ^1^Department of Bioengineering and Hillman Cancer Center, University of Pittsburgh, Pittsburgh, Pennsylvania, United States; ^2^McGowan Institute of Regenerative Medicine, University of Pittsburgh, Pittsburgh, Pennsylvania, United States

**Keywords:** chemotherapy, macrophage, mesothelial, microenvironment, ovarian cancer

## Abstract

The metastatic ovarian cancer microenvironment is characterized by an intricate interaction network between cancer cells and host cells. This complex heterotypic cancer-host cell crosstalk results in an environment that promotes cancer cell metastasis and treatment resistance, leading to poor patient prognosis and survival. In this review, we focus on two host cell types found in the ovarian cancer microenvironment: mesothelial cells and tumor-associated macrophages. Mesothelial cells make up the protective lining of organs in the abdominal cavity. Cancer cells attach and invade through the mesothelial monolayer to form metastatic lesions. Crosstalk between mesothelial and cancer cells can contribute to metastatic progression and chemotherapy resistance. Tumor-associated macrophages are the most abundant immune cell type in the ovarian cancer microenvironment with heterogeneous subpopulations exhibiting protumor or antitumor functions. Macrophage reprogramming toward a protumor or antitumor state can be influenced by chemotherapy and communication with cancer cells, resulting in cancer cell invasion and treatment resistance. A better understanding of cancer-mesothelial and cancer-macrophage crosstalk will uncover biomarkers of metastatic progression and therapeutic targets to restore chemotherapy sensitivity.

## Graphical abstract

**Figure:**
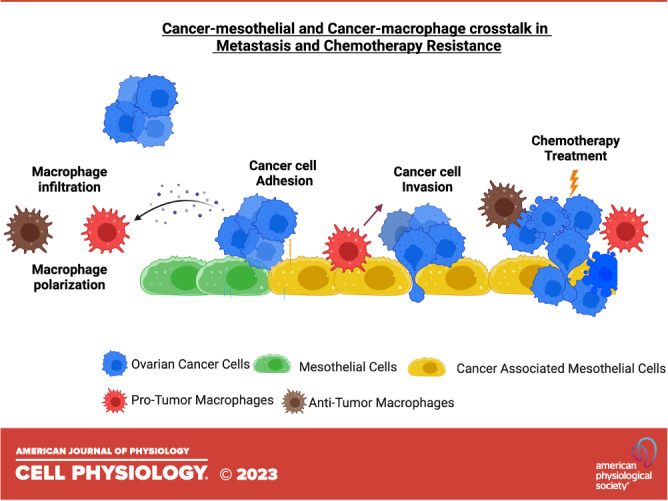

## INTRODUCTION

Ovarian cancer is the deadliest form of cancer compared with other gynecological malignancies (1). In the United States, a total of 19,710 new ovarian cancer cases and 13,270 deaths are estimated in 2023 (1) with a five-year survival estimate below 50% (1, 2). Ovarian cancer is typically diagnosed at an advanced stage, with 23% of patients having regional metastases and 48% of patients having distant metastases (1). Platinum-based chemotherapy represents the first line of treatment for ovarian cancer (3). In the metastatic setting, even with debulking surgery and aggressive chemotherapy, most patients will still experience disease recurrence (4–6). Therefore, there is a crucial need to understand the mechanisms of chemoresistance and design novel treatments.

During metastatic progression and response to chemotherapy, ovarian cancer cells interact with host cells in the tumor microenvironment (TME) within a three-dimensional (3-D) extracellular matrix. Host cells abundant in ovarian TME include tumor-associated macrophages (TAMs), fibroblasts, T cells, endothelial cells, adipocytes, and mesothelial cells (7). Importantly, heterotypic cancer-host interactions have been shown to contribute both to metastatic progression (4, 8–10) and chemotherapy treatment responses (11). In this review, we discuss the interactions of mesothelial cells and macrophages with ovarian cancer cells in the context of metastasis and chemotherapy resistance. Cancer cells attach and migrate through the mesothelial barrier of abdominal organs to establish metastatic implants (5, 12–14). Mesothelial cells have been shown to promote an invasive and prosurvival cancer cell phenotype through paracrine and juxtracrine signaling (12, 14). Macrophages recruited to the TME convert into tumor-associated macrophages (TAMs) and can be classified into subpopulations along a spectrum with differential effects on tumor progression (15). On one end of the spectrum, protumor macrophages have been shown to enhance ovarian cancer cell invasion, stemness, and chemotherapy resistance. On the other end of the spectrum, antitumor macrophages have been linked with augmented cytotoxic T-cell function and improved response to immunotherapy (15, 16). Elucidating cancer-mesothelial and cancer-macrophage signaling is critical in discovering novel TME-focused mechanisms for prognostic and therapeutic use that can ultimately improve patient outcomes.

## MESOTHELIAL CELLS

### Cancer-Mesothelial Interactions That Promote Ovarian Cancer Metastasis

**Figure 1.**
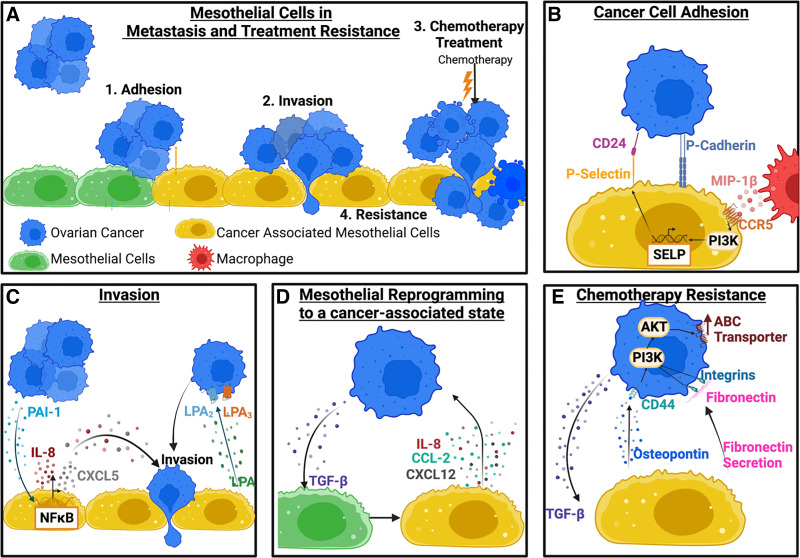
Mesothelial-cancer cell interactions in the metastatic cascade and chemotherapy resistance. *A*: ovarian cancer spheroids (blue) adhere to the mesothelial lining (green), followed by invasion into the underlying tissue. Mesothelial cells reprogrammed by cancer cells become cancer-associated (yellow) and can impact chemotherapy responses. *B*: cancer cell adhesion to mesothelial cells is mediated via CD24 expression on the cancer cells interacting with P-selectin. P-selectin expression is increased by macrophage-secreted MIP-1β that binds to CCR5 on mesothelial cells and activates PI3K signaling. *C*: ovarian cancer cells secrete PAI-1 that activates NF-κB signaling in mesothelial cells to induce cancer-associated reprogramming (yellow). These cancer-associated mesothelial cells secrete IL-8 and CXCL-5 to increase cancer cell invasion. Another mechanism of mesothelial-induced ovarian cancer invasion via LPA secretion. *D*: mesothelial reprogramming is caused by the release of TGF-β from cancer cells. In turn, mesothelial cells increase secretion of CXCL-12, CCL-2, and IL-8 to promote cancer cell metastatic potential. *E*: chemotherapy resistance can be mediated via mesothelial-derived osteopontin and fibronectin. Osteopontin interacts with CD44 on cancer cells, which activates prosurvival PI3K and AKT signaling resulting in chemoresistance. LPA, lysophosphatidic acid. Created with BioRender.com.

Mesothelial-cancer cell cross talk plays a critical role in multiple steps of the metastatic cascade including cancer cell adhesion, spreading, and invasion (Fig. 1*A*). Expression of transmembrane proteins used in heterotypic cell-cell adhesion critically impacts the ability of ovarian cancer cells to attach to the peritoneal wall (Fig. 1*B*). For example, P-cadherin, a transmembrane protein expressed on ovarian cancer cells, has been shown to promote adhesion of cancer cells to mesothelial cells (17). P-selectin is another transmembrane protein expressed on mesothelial cells and has been shown to be upregulated after coculture with TAMs (18). Increased expression of P-selectin was induced through CCR5 and PI3K signaling activation due to TAM-secreted MIP-1β, resulting in higher SELP (encodes for P-selectin) transcription (Fig. 1*B*). Ovarian cancer cells attach to the mesothelial monolayer via the P-selectin ligand CD24 (18). In addition to alterations in the TME that impact cell surface receptor expression in mesothelial cells, it has been shown that pyruvate dehydrogenase kinase 1 (PDK1) overexpression in ovarian cancer cells results in increased adhesion to mesothelial cells via α5β1 integrins and secretion of IL-8 (19).

Hyaluronan (HA, hyaluronic acid) is a critical component of the extracellular matrix in the ovarian tumor microenvironment and has been shown to coat the mesothelial cell surface. HA interacts with the CD44 receptor on tumor cells to promote cell adhesion in vitro via a mechanism that involves receptor glycosylation (20). Another in vitro study investigated HA cell surface levels in tumor cell subpopulations and observed that HA levels determine adhesion potential to mesothelial cells (21). A histopathological study in patient samples with ovarian cancer revealed that high CD44 expression correlates with tumor stage, grade, histological subtype, and poorer survival outcomes (22). In addition, analysis of HA localization in the TME revealed two staining patterns: *1*) enrichment of pericellular HA expression in tumor cells and *2*) expression of HA only in stromal cells within the tumor. However, only pericellular tumor cell HA expression was an independent unfavorable prognostic marker for patient survival (23). Once cancer cells adhere to the peritoneal wall, they can spread on mesothelial cells, migrate through the mesothelial barrier, and invade the underlying tissue (Fig. 1*C*). Ovarian cancer cells secrete plasminogen activator inhibitor-1 (PAI-1) into the TME to facilitate transmesothelial migration (24). Under two-dimensional (2-D) culture conditions, mesothelial cells exposed to cancer-derived PAI-1 upregulate IL-8 and CXCL-5 secretion in an NF-κB-dependent mechanism that promotes ovarian cancer cell invasion via bidirectional cell-cell signaling (24). Intrinsic expression of the cell membrane-localized protein CD157 in ovarian cancer cells has been shown to promote transmesothelial migration (25), whereas similar prometastatic effects have been demonstrated with overexpression of the tumor-secreted peptidase kallikrein-related-peptidase 4 (KLK4) (26). In addition, mesothelial cells have been shown to secrete lysophosphatidic acid (LPA), which can stimulate ovarian cancer migration (27). Blocking the production of LPA in mesothelial cells halts ovarian cancer migration in transwell assays (27).

Exosomes and extracellular vesicles represent another critical regulator of paracrine signaling in the tumor microenvironment. CD44 transfer from tumor cells to mesothelial cells via exosomes results in increased MMP-9 secretion by mesothelial cells that induces ovarian cancer invasion in vitro (28). This mechanism has also been reported in clinical samples, where CD44+ mesothelial cells are located near the tumor-invasive front (28). Exosomal annexin A1 (ANXA2) represents another example of a tumor-derived factor that activates PI3K/AKT/mTOR signaling in mesothelial cells to promote mesothelial-to-mesenchymal transition and MMP-2/-9 upregulation (29). Furthermore, miR-99a-5p has been found to be elevated in cancer-derived exosomes using microarray and serum analyses in patients with epithelial ovarian cancer (EOC) (30). Transfer of these exosomes to mesothelial cells or transfection with only miR-99a-5p increased expression of fibronectin and vitronectin, resulting in enhanced cancer cell invasion across transwells plated with mesothelial barriers (30). An in-depth discussion of exosomes, miRNA, and circular and noncoding RNA in peritoneal metastasis is described in another review (31).

Paracrine interactions with cancer cells induce mesothelial cell reprogramming toward a cancer-associated mesothelial state and can further potentiate metastasis (Fig. 1*D*). Cancer-associated mesothelial cells express heparinase, resulting in the degradation of heparan sulfate proteoglycans, which has been linked to tumor stage (32). Exposure to ascites, the fluid accumulating in the abdominal cavity during advanced disease, increases the expression of MUC16 in mesothelial cells in vitro (33) MUC16 encodes for CA125, a biomarker for ovarian cancer progression (33). Ascites-induced activation of MUC16 is independent of NF-κB, however, AKT regulates the expression and shedding of MUC16 (33). Furthermore, cancer-associated mesothelial cells overexpress paracrine factors that can promote cancer invasion. For example, mesothelial cells treated with transforming growth factor beta (TGF-β) increase the secretion of CCL-2, IL-8, and CXCL-12 (34). In turn, cancer cells exposed to cancer-associated mesothelial cell-derived CCL-2 show increased migration and transmesothelial migration under 2-D culture conditions (34). Furthermore, CCL-2 activates the P38-MAPK pathway in cancer cells, and high expression of the CCL-2 receptor CCR-2 has been shown to be a prognostic marker for overall and progression-free survival in ovarian cancer (34). Mesothelial cells exposed to ovarian cancer-conditioned medium in vitro have also been shown to constitutively express the ET-1 and the receptors ETaR and ETbR (35). ET-1 upregulates SNAIL, Tenascin-C, Vimentin, and reduces E-cadherin, resulting in a mesothelial-to-mesenchymal transition, which supports cancer progression (35). Overall, these studies demonstrate the complex roles that mesothelial cells play in ovarian cancer metastatic dissemination and how cancer-induced mesothelial cell reprogramming can further potentiate cancer aggressiveness (Fig. 1, *A*–*D*).

### Cancer-Associated Mesothelial Cells Induce Chemotherapy Resistance

Following treatment with chemotherapy, mesothelial cells exhibit similar drug uptake as tumor cells and engage DNA damage response pathways (36). Mesothelial-derived deposition of TGF-βI within the extracellular matrix via a secreted protein acidic and rich in cysteine (SPARC)-dependent mechanism has been shown to regulate ovarian cancer cell apoptosis following treatment with paclitaxel (37). Furthermore, expression of the adhesion molecule VCAM-1 in mesothelial cells has been correlated with tumor stage in the clinic and platinum resistance in preclinical ovarian cancer models (38, 39). In vitro studies of cancer cells cocultured with TGF-β-conditioned mesothelial cells revealed activation of the PI3K/AKT/mTOR survival pathway that blunted the cytotoxic effects of platinum chemotherapy (5). In addition, osteopontin released by cancer-associated mesothelial cells has been shown to induce chemoresistance via activation of CD44, PI3K-AKT signaling, and ABC drug transporter in ovarian cancer cells in vitro (40). This mechanism involves a bidirectional feedback loop, where TGF-β secreted by cancer cells induces mesothelial cell transition toward a cancer-associated state and higher levels of osteopontin. Inhibition of osteopontin in vivo using neutralization antibodies enhanced response to cisplatin in both human and mouse xenografts (40). Approaches that involve targeting prosurvival PI3k-AKT signaling in cancer cells or the TGF-β-dependent mesothelial reprogramming hold promise for restoring chemotherapy sensitivity. Furthermore, direct heterotypic cell-cell contact can also modulate chemotherapy sensitivity via overexpression of the P-glycoprotein multidrug resistance transporter in cancer cells (41). Hence, a better understanding of how cancer-mesothelial crosstalk regulates chemotherapy response will yield new therapeutic targets for restoring drug sensitivity (Fig. 1*E*).

Many of the signaling events (Fig. 1) described earlier involve bidirectional paracrine communication, where cancer-associated mesothelial cells secrete proinvasive (e.g., IL-8) and prosurvival (e.g., fibronectin) factors to support ovarian cancer metastatic progression. These interactions also involve exchange of exosomes, binding to ECM (e.g., HA), juxtracrine cancer-mesothelial cell contact, and crosstalk with other cell types (e.g., TAMs) that depend on the cancer genetic makeup (18, 24). Future studies that use a systems biology approach to map the myriad of microenvironmental factors on converging pathways in cancer cells (e.g., NF-κB, PI3K-ΑΚΤ) and to identify context-dependent targets hold promise for halting ovarian cancer progression.

## MACROPHAGES

Tumor-associated macrophages are diverse and respond to multiple factors in the tumor microenvironment that regulate their functional state. The TME complexity results in TAM subpopulations with differential effects on tumor progression. Many studies use cell surface markers, including CD163, CD204, and CD206 to describe TAMs with a protumor function, whereas CD80, CD86, iNOS, and HLA-DR denote TAMs with an antitumor function (15, 16). However, direct analysis in patient samples with ovarian cancer has shown a complex picture with TAMs overexpressing both CD163 and CD86 markers compared with tumor-naïve macrophages (42). Hence, multiparametric single-cell analysis methods combined with functional readouts (e.g., cytokine secretions, cancer invasion) are necessary to accurately define TAM subpopulation effects on cancer cells (43).

### Treatment-Induced Macrophage Infiltration into the Tumor Microenvironment

Macrophage infiltration is highly dependent on the subtype of ovarian cancer, the method of treatment, and the elements constituting the TME. Due to the wide range of phenotypes and functions of TAMs in ovarian cancer, their infiltration into the TME can be beneficial or detrimental to tumorigenesis and response to treatment (44). Macrophage infiltration behaviors and the effect of anticancer treatments can be studied through characterization of cells directly from patient samples, in vivo, and in vitro model systems. Histological analysis of ovarian tumors showed increased foamy and hemosiderin-laden macrophages after neoadjuvant chemotherapy, as well as larger degrees of fibrosis and calcification of tumors with atypical cellular morphology (45–47). Another study compared macrophage infiltration in high-grade serous ovarian carcinoma (HGSOC), clear cell carcinoma (CCC), and endometrioid carcinoma (EC) and found that HGSOC had the highest TAM infiltration (48). Receptor-binding cancer antigen (RCAS1) expression in cancer cells is linked to an immunosuppressive TME, and it was demonstrated that cytoplasmic RCAS1 expression in ovarian tumor samples correlated with a larger degree of macrophage infiltration (49). However, another histological analysis found a heterogenous pattern in the infiltration of macrophages between matched pre- and postneoadjuvant chemotherapy tumor samples, with only a subset of patients exhibiting an increase in PD-L1+CD68+ macrophage density (50). Studies using flow cytometry-based analysis of TAMs in ascites have shown that patients with a higher expression of the protumor CD163+ marker exhibit shorter relapse-free survival (42). Histological profiling studies that evaluated the absolute number of CD163+positive cells have reported mixed results. One study showed that the number of CD163+positive cells or the ratio of CD163+/CD68+ cells predicted poor survival, but the same did not hold true for the pan-macrophage marker CD68+ (51). Another study found no association between the number of CD163+positive cells with survival (52). These discrepancies in predicting overall survival could be due to the lack of considering the fraction of antitumor TAMs (HLA-DR+ and iNOS+) that support antitumor immunity and can vary significantly between patient groups (53). Furthermore, it is critical to consider the location of TAMs in the tumor microenvironment. By using a digital pathology approach, the density of stromal CD68+ macrophages was found to predict good survival outcomes, whereas high intraepithelial CD68+ macrophages were not predictive of survival (54). These results are consistent with another study showing no association between total CD68+ cells and patient survival (55). To comprehensively evaluate the association of TAMs with clinical outcomes in different patient cohorts, a meta-analysis of TAM subpopulations and clinical outcomes was conducted and showed a consistent association between macrophage infiltration and tumor stage across multiple cohorts, whereas the association of TAMs and specifically protumor CD163+ TAMs with survival was cohort dependent (56). Importantly, across all cohorts, the ratio of antitumor HLA-DR+ TAMs to protumor CD163+ TAMs was more strongly associated with survival compared with absolute TAM numbers (56). Taken together, these studies highlight the heterogeneity of TAM subpopulations in ovarian cancer and the need to simultaneously track abundance and balance between pro- and antitumor TAM subsets in a framework that also employs functional studies.

**Figure 2.**
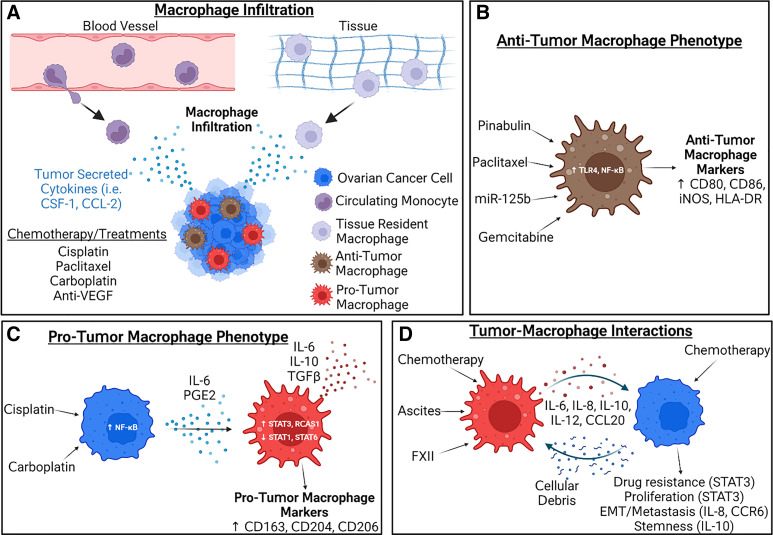
Macrophage infiltration, reprogramming, and interactions with ovarian cancer cells. *A*: macrophages recruited to the ovarian tumor microenvironment become tumor associated. Circulating monocytes or tissue-resident macrophages are recruited in response to tumor cell-secreted cytokines (e.g., CSF-1 and CCL-2). Chemotherapy (e.g., cisplatin, paclitaxel) and other targeted therapies such as anti-VEGF therapy can differentially impact macrophage infiltration. *B*: treatments such as pinabulin, paclitaxel, gemcitabine, and miR-125b upregulate TLR4 and NF-κB expression in macrophages, resulting in a protumor macrophage phenotype. This phenotype is characterized by increased surface expression of CD80 and CD86. *C*: chemotherapies such as cisplatin and carboplatin upregulate NF-κB expression in tumor cells, resulting in increased expression of IL-6 and PGE2. This increases STAT3 and RCAS1 expression and decreases STAT1 and STAT6 in macrophages, resulting in an antitumor macrophage phenotype. This phenotype is characterized by increased IL-6, IL-10, and TGF-β secretion and surface expression of CD163 and CD206. *D*: positive signaling feedback loop between macrophages and ovarian cancer cells can be modulated by chemotherapy and ascites. Cellular debris released by dying cancer cells and IL-6, IL-8, IL-10, IL-12, and CCL-20 secretion by macrophages, results in protumorigenic cancer cell phenotypes (e.g., increased proliferation, invasion, and stemness). Created with BioRender.com.

In vivo and in vitro models can also be used to study macrophage infiltration behaviors seen in the complex TME and investigate how a variety of treatments impact macrophage recruitment and tumor cell response. Depletion of CD163 + Tim4+ macrophages in a mouse model of orthotopic ovarian cancer demonstrated the critical role of these protumor TAMs in promoting the malignant progression of ovarian cancer (57). In another study with mouse models, CD163+ TAM numbers were shown to increase with estrogen treatment in the TME, especially in hypoxic and necrotic regions (58). Hypoxia plays a role in macrophage infiltration and the formation of new blood vessels (angiogenesis) (59). In mouse models, administration of the angiogenesis-targeted anti-VEGF antibody and paclitaxel led to treatment resistance, but the addition of a CSF-1R inhibitor (CSF-1 regulates macrophage survival and migration) restored sensitivity to anti-VEGF therapy and decreased macrophage infiltration and tumor burden (59). Macrophage infiltration in vivo is also impacted by the treatment schedule. In this preclinical study, a dose-dense cisplatin and paclitaxel treatment protocol resulted in higher F4/80+ macrophage recruitment to the TME compared with the maximum tolerated dose protocol (60). In addition, targeting macrophages with clodronate liposome reduced chemotherapy treatment response (60). In an in vitro study of chemotherapy response, it was shown that ovarian cancer cells treated with paclitaxel and carboplatin upregulate CCL-2 (61). This increase in CCL-2 was also seen in an in vivo mouse model, but there was no significant difference in macrophage infiltration (61). These studies demonstrate that macrophage infiltration in vivo depends on the subtype of ovarian cancer, chemotherapeutic treatment schedule, and TME composition (e.g., oxygen tension) (Fig. 2*A*).

### Macrophage Reprogramming Due to Chemotherapy

It is well established that tumor-secreted factors in the TME promote the reprogramming of macrophages toward a protumor phenotype (16). One study demonstrated that coculture of ovarian cancer cells and macrophages increased the secretion of multiple secreted factors, resulting in increased CD204 and CD206 marker expression (62). In particular, cytokines, chemokines, and MMPs that are known to be present in the TME (including CSF-1, IL-13, CCL-5, CCL-22, MMP-7, MMP-9, VEGF, and TNF-α) were found to be increased in the conditioned medium (62). Another in vitro coculture study showed the heterogeneous ability of different ovarian cancer cell lines to increase CD163 expression in primary monocyte-derived macrophages (63). Chemotherapy can also directly affect macrophage phenotypes or alter tumor cell-macrophage crosstalk that results in changes in macrophage activation state. In a study analyzing patient samples, platinum-based neoadjuvant chemotherapy reduced total macrophage numbers from the TME but enhanced the antitumor abilities of remaining macrophages (64). However, carboplatin decreased, and paclitaxel increased the number of CD163+ and CD206+ TAMs in a mouse model, demonstrating the specific effects of chemotherapeutic agents on macrophage reprogramming (64). Plinabulin is a microtubule-destabilizing agent that was shown to increase antitumor CD80+ and CD86+ TAMs in patient samples after only 48 h of treatment (Fig. 2*B*; 65). In vitro, plinabulin increased proliferation of CD80+ and CD86+ TAMs, but not CD163+ and CD206+ TAMs (65). Another study reported that paclitaxel induces TLR4 and NF-κB signaling in macrophages, resulting in increased TNF-α, IL-12, and nitrite secretions, which is evidence of an antitumor TAM phenotype (Fig. 2*B*) (66). In another study, treating cancer cells with platinum-based chemotherapy increased their secretion of IL-6 and prostaglandin E2 (PGE2) through an NF-κB mediated pathway (67). Here, chemotherapy-treated cancer cells increased the expression of protumor CD206 and CD163 markers on TAMs, with increased IL-10 secretion and STAT3 activation (Fig. 2*C*) (67). These TAMs were found to have an increased sensitivity to chemotherapy treatment (67). As stated earlier, RCAS1 is linked to immunosuppression and a protumor TAM phenotype. Higher levels of RCAS1 were detected in macrophages in the central part of the tumors of patients who received neoadjuvant chemotherapy compared with those who received cytoreductive surgery (68). Τhese heterogeneous chemotherapy effects on TAM subsets can be explained by the indirect effects of platinum agents on macrophages via exposure to cancer cell-derived factors (e.g., IL-6) that promote protumor TAM conversion (64). On the other hand, antimitotic therapies can directly activate the TLR4/NF-κB/JNK signaling axis in macrophages and drive expression of antitumor markers (e.g., CD80) (66). Therefore, further studies are necessary to investigate how chemotherapies modulate transition between protumor and antitumor TAM states, as well as the phenotype of newly recruited macrophages. These studies should also consider the genetic background of the tumor cells (e.g., DNA damage repair efficiency and intrinsic chemotherapy sensitivity), as well as potential interactions with stromal cells (e.g., mesothelial cells as described in the following section).

A clinical trial in patients with platinum-resistant ovarian cancer combined gemcitabine (chemotherapy), Pegintron (IFN-γ), and a p53 synthetic long peptide vaccine (69). This study showed that gemcitabine treatment alone was sufficient to increase the fraction of CD163− TAMs in the TME, and the combination of all therapeutics increased T-cell responses (69). Hyaluronic acid-based nanoparticles encapsulating miR-125b were shown to be taken up by macrophages in vivo, resulting in increased number of antitumor CD80+ TAMs and decreased number of protumor CD206+ TAMs in the TME (70). miR-125b has been shown to inhibit interferon regulatory factor 4 (IRF4) in macrophages in a mechanism that upregulates expression of MHC-II, CD80, and CD86 to promote macrophage costimulatory function and potentiate adaptive immunity (71). Combined treatment of these miR-125b-containing nanoparticles with paclitaxel further reduced the ratio of protumor to antitumor TAMs and the accumulation of ascitic fluid in the peritoneal cavity (70). Natural compounds, such as cardamonin, represent another approach to suppress protumor TAM function in ovarian cancer (72). In vitro studies showed that treatment with cardamonin reduced the expression of protumor CD206 and increased the expression of antitumor CD86 in TAMs via a mechanism involving mTOR-regulated STAT3 activity (72). Hence, to develop rational combination therapies, it will be essential to understand the effect on macrophage survival, phenotype, and chemotherapy treatment responses in the TME in a time- and context-dependent manner.

Lastly, crosstalk between ovarian cancer cells and macrophages has been shown to influence the metabolism of both cell types and macrophage activation state. One study showed that hyaluronic acid (HA) secreted from ovarian cancer cells can promote membrane cholesterol efflux and the depletion of cholesterol-rich membrane microdomains in macrophages. This caused an increase in IL-4 signaling and a decrease in IFN-γ signaling, indicative of TAMs with protumor phenotypes (73). Consistent with this preclinical finding, IL-4 levels in ascites have been shown to be upregulated in a subset of patients and can be coexpressed with other anti-inflammatory cytokines such as IL-10 that may further contribute to reprogramming of macrophages toward a protumor phenotype (74). Another study showed that ovarian cancer cells with low glutamine synthase and high glutaminolysis become addicted to extracellular glutamine, resulting in the release of N-acetyl aspartate (NAA). Increased NAA, alongside extracellular IL-10, causes macrophages to have increased glutamine synthase expression and increased CD163 and CD206 expression (75). Further research is required to elucidate the complex metabolic coupling between ovarian cancer cells and macrophages.

### Tumor-Macrophage Interactions: Effects on Cancer Cell Phenotype

The presence of TAMs in the TME have diverse effects on the metastatic capability, stemness, and treatment response in ovarian cancer (15). It has been shown that there are increased CD163+ TAMs in the malignant ascites of patients with ovarian cancer (76). Ascites from patient samples caused STAT3 activation in macrophages in vitro, resulting in increased IL-6 and IL-10 secretion by macrophages (Fig. 2*D*) (76). Furthermore, ascites and macrophages increased STAT3 activation in ovarian cancer cells, which is associated with tumor cell chemoresistance and proliferation (76). High levels of coagulation factor XII (FXII) have been found in the peritoneum of patients with ovarian cancer. Exposing macrophages to this factor increased CD163, IL-10, CCL-18, IL-8, and CXCR2 expression (77). Ovarian cancer cells increased invasiveness when subjected to conditioned media from FXII-stimulated macrophages (77). A coculture model showed that endothelial cells increase migration, tube formation, and IL-8 expression when exposed to TAM and ovarian cancer-conditioned media, indicating increased angiogenesis (78).

Treatment with chemotherapy can further alter tumor cell-macrophage interactions. Taxane and platinum-based chemotherapies were shown to cause the release of cellular debris from ovarian cancer cells, which stimulated proinflammatory cytokine and bioactive lipid secretion from macrophages (79). This created a protumorigenic and prometastatic TME (79). Another study demonstrated that cisplatin-treated macrophages increase CCL-20 production, which activates CCR6 on ovarian cancer cells to promote EMT and subsequent migration (80). An in vitro model showed that cisplatin-sensitive ovarian cancer cells experienced significant gene expression changes related to stemness, EMT, and drug resistance after treatment with chemotherapy and coculture with macrophages. In addition, there was an increase in transcription of macrophage markers related to a protumor phenotype after coculture, including CD163, IL-10, and CCL-22 (81). Ovarian cancer stem cells and macrophages grown together in spheroids result in increased CD206, CD163, and IL-10 expression, as well as WNT signaling pathway activity, compared with ovarian cancer cells grown in isolation (82). Spheroids formed using ovarian cancer stem cells were less sensitive to carboplatin chemotherapy, maintained stemness, and were more invasive in transwell assays (82).

Magnetic and therapeutic nanoparticles, which can be preferentially taken up by macrophages compared with tumor cells, have also been used for modulating macrophage activation state and chemotherapy delivery (83). Monophosphoryl lipid A (MPLA) and interferon-γ (IFN-γ) injection in a mouse model resulted in type I IFN signaling, which promoted an increase in iNOS+ TAMs and a decrease in CD206+ TAMs (84). This increased macrophage secretion of IL-12 and TNF-α with augmented cytotoxic T-cell function, causing an improved response to chemotherapy, reduced tumor burden and prolonged survival (84). Lastly, macrophage and tumor cell interactions can modulate the response to immunotherapies (85). Molecular profiling of patients showed that individuals with a higher density of antitumor TAMs have better responses to PD-1 immune checkpoint blockade, whereas those with higher protumor TAM density are more resistant to this type of immunotherapy (86). Understanding the effects of macrophages on tumor cell prosurvival signaling and metastatic progression, especially in response to therapy, will help lead to the development of new therapies that address multiple components of the TME.

## MESOTHELIAL-MACROPHAGE INTERACTIONS IN THE OVARIAN CANCER MICROENVIRONMENT

Despite the important roles of mesothelial cells and macrophages in ovarian cancer TME, few studies have investigated mechanisms of mesothelial-macrophage crosstalk. A study that used genetically engineered mouse models identified an LYVE1+ subpopulation of macrophages that was localized near peritoneal mesothelial cells. These macrophages exhibit prometastatic behaviors driven by CSF-1 secretion by WT1+ fibroblasts and mesothelial cells (87). In support of these findings, additional research has shown that CSF-1 secreted by mesothelial cells maintains homeostasis of peritoneal macrophages (88). Another study on the paracrine effects of tumor-derived factors revealed that SPARC overexpression in cancer cells decreased macrophage and mesothelial production of IL-6, MMPs, urokinase plasminogen activator, prostaglandin E2, and 8-isoprostanes (89). These secreted factors are critical regulators of ovarian cancer-associated inflammation and metastasis. In addition, SPARC was found to reduce CCL-2 production and macrophage chemotaxis (89).

As described in *Cancer-Associated Mesothelial Cells Induce Chemotherapy Resistance* above, SPARC expression in mesothelial cells caused an increase in TGF-βI secretion (37). Interestingly, macrophages have also been shown to secrete TGF-βI during interaction with p53 mutant fallopian tube epithelial cells to establish an immunosuppressive microenvironment (90). Targeting TGF-βI in a mouse model using a neutralization antibody reduced peritoneal tumor size and increased the number of CD45+ CD3+ CD4− CD8− T cells (90). Hence, these complex tumor-mesothelial-macrophage paracrine loops can also be exploited therapeutically to develop new combination therapies that block the tumor-permissive functions of the ovarian TME (5, 37–40, 90).

Furthermore, TAM secreted MIP-1β-activated CCR5/PI3K signaling in mesothelial cells, upregulating P-selectin expression on the mesothelial monolayer and enhancing tumor cell adhesion via CD24 (18). The HA receptor CD44 has also been shown to be upregulated in both mesothelial cells and macrophages isolated from the ascites of patients with ovarian cancer compared with benign ascites (91). High CD44 expression is indicative of an aggressive phenotype, however, this study did not evaluate the interaction between these two cell types. Another study investigated the impact of obesity on ovarian cancer metastasis and found a decreased population of antitumor iNOS+ TAMs in the TME of obese mice (92). In addition, it was discovered that there were increased microvilli on mesothelial cells, suggesting increased tumor cell adhesion to the mesothelial monolayer. Further research could elucidate if macrophage-mesothelial crosstalk is responsible for these protumoral changes in the TME (92). Lastly, the developmental patterning gene HOXA9 has been implicated in encouraging protumor phenotypes in peritoneal macrophages and implantation of tumor cells to the mesothelial monolayer, highlighting the importance of these cells in the TME and providing another potential connection between the two cell types (93). Taken together, these studies demonstrate the importance of mesothelial-macrophage interactions in establishing a prometastatic ovarian cancer TME.

## CONCLUSIONS AND FUTURE DIRECTIONS

The complex crosstalk between ovarian cancer cells and other host cells in the TME is an essential part of metastatic progression and response to chemotherapy. Hence, studies which further our knowledge of heterotypic signaling mechanisms are essential to reducing the mortality from ovarian cancer and earlier detection of metastasis. Mesothelial cells contribute to both the metastatic cascade and chemotherapy resistance by promoting an aggressive cancer phenotype and prosurvival signaling. The heterogeneity of mesothelial cells in different metastatic sites and mechanisms by which cancer cells reprogram mesothelial cells remain elusive. Furthermore, it will be critical to dissect the impact of chemotherapy on mesothelial cells and their interactions with other host cells in the complex TME. Macrophages recruited in the TME convert to a TAM state that enhances cancer cell invasiveness and impacts response to chemotherapy. Future studies are needed on deep phenotyping of TAM subpopulations enriched following chemotherapy, tracking the dynamics of TAM state switching and infiltration, and how TAMs interact with other immune cells (e.g., cytotoxic and regulatory T cells) to shape ant-tumor immunity. The development of in vitro models that allow for spatial and temporal regulation of cell localization and signaling in 3-D extracellular matrices, as well as bioinformatic approaches to identify biomarkers and dysregulated pathways, are crucial for drug screening in a physiologically relevant environment. In sum, a better understanding of cancer-mesothelial and cancer-macrophage cross talk in the ovarian TME will move research one step closer to discovering microenvironment-focused biomarkers and developing novel treatment options.

## GRANTS

This work was supported by the US National Institutes of Health (R00 CA222554 to I.K.Z.), the Elsa Pardee Foundation (pilot grant to I.K.Z.), a BIRM T32 Fellowship (NIBIB/NIH T32 EB003392 to D.E.J.), a Magee Women’s Research Institute Ovarian Cancer pilot award, the UPMC Hillman Cancer Center and the Department of Bioengineering, School of Engineering at the University of Pittsburgh.

## DISCLOSURES

No conflicts of interest, financial or otherwise, are declared by the authors.

## AUTHOR CONTRIBUTIONS

D.E.J. and D.G.K. prepared figures; D.E.J. and D.G.K. drafted manuscript; D.E.J., D.G.K., and I.K.Z. edited and revised manuscript; D.E.J., D.G.K., and I.K.Z. approved final version of manuscript.
